# Assessment of immunization data management practices in Cameroon: unveiling potential barriers to immunization data quality

**DOI:** 10.1186/s12913-023-09965-9

**Published:** 2023-09-27

**Authors:** Yauba Saidu, Jessica Gu, Budzi Michael Ngenge, Sangwe Clovis Nchinjoh, Amani Adidja, Nadege Edwidge Nnang, Nkwain Jude Muteh, Vouking Marius Zambou, Clarence Mbanga, Valirie Ndip Agbor, Diaby Ousmane, Andreas Ateke Njoh, Junie Flegere, Demba Diack, Owens Wiwa, Emmanuele Montomoli, Sue Ann Costa Clemens, Ralf Clemens

**Affiliations:** 1 Clinton Health Access Initiative Inc, PO Box 2664, Yaounde, Cameroon; 2 Institute for Global Health, University of Siena, Siena, 53100 Italy; 3 Global Vaccine Team, Clinton Health Access Initiative Inc, Boston, MA 02127 USA; 4 Faculty of Medicine and Biomedical Sciences, University of Yaoundé 1, Yaoundé, Cameroon; 5 Gavi, The Vaccine Alliance, Geneva, Switzerland; 6 UNICEF, Yaoundé, Cameroun; 7 Clinical Trial Service Unit and Epidemiological Studies Unit (CTSU), Nuffield Department of Population Health, University of Oxford, Oxford, UK; 8 Department of Projects, Ministry of Public Health, Yaounde, Cameroon; 9 Expanded Program on Immunization, Ministry of Public Health, PO Box 2084, Yaoundé, Cameroon; 10 School of Global Health and Bioethics, Euclid University, PO Box 157, Bangui, Central African Republic; 11 Department Molecular Medicine, University of Siena, Via Aldo Moro 3, 53100 Siena, Italy; 12 VisMederi srl, Via Ferrini 53, 53035 Siena, Italy; 13 Department of Pediatrics, University of Oxford, Oxford, UK

**Keywords:** Data management, Practices, Data quality, Expanded program of immunization, Vaccination staff

## Abstract

**Background:**

One crucial obstacle to attaining universal immunization coverage in Sub-Saharan Africa is the paucity of timely and high-quality data. This challenge, in part, stems from the fact that many frontline immunization staff in this part of the world are commonly overburdened with multiple data-related responsibilities that often compete with their clinical tasks, which in turn could affect their data collection practices. This study assessed the data management practices of immunization staff and unveiled potential barriers impacting immunization data quality in Cameroon.

**Methods:**

A descriptive cross-sectional study was conducted, involving health districts and health facilities in all 10 regions in Cameroon selected by a multi-stage sampling scheme. Structured questionnaires and observation checklists were used to collect data from Expanded Program of Immunization (EPI) staff, and data were analyzed using STATA VERSION 13.0 (StataCorp LP. 2015. College Station, TX).

**Results:**

A total of 265 facilities in 68 health districts were assessed. There was limited availability of some data recording tools like vaccination cards (43%), maintenance registers (8%), and stock cards (57%) in most health facilities. Core data collection tools were incompletely filled in a significant proportion of facilities (37% for registers and 81% for tally sheets). Almost every health facility (89%) did not adhere to the recommendation of filling tally sheets during vaccination; the filling was instead done either before (51% of facilities) or after (25% of facilities) vaccinating several children. Moreso, about 8% of facilities did not collect data on vaccine administration. About a third of facilities did not collect data on stock levels (35%), vaccine storage temperatures (21%), and vaccine wastage (39%).

**Conclusion:**

Our findings unveil important gaps in data collection practices at the facility level that could adversely affect Cameroon’s immunization data quality. It highlights the urgent need for systematic capacity building of frontline immunization staff on data management capacity, standardizing data management processes, and building systems that ensure constant availability of data recording tools at the facility level.

## Introduction

Estimates suggest that between 2010 and 2019, immunization alone has prevented over 37 million deaths globally [1, 2]. Moreso, immunization brings added value to communities by reducing families’ health expenditures and improving countries’ productivity and resilience. For example, some studies have reported that immunization returns an estimated US$16 for every US$ 1 invested, and this return can reach US$44 when broader social and economic benefits or vaccination are considered [3, 4]. But despite these benefits, national and subnational vaccination coverage rates have plateaued in several countries, particularly those in sub-Saharan Africa (SSA), where coverage rates for basically all antigens are considerably lowered compared to other regions [1].

One key contributing factor to the above phenomenon is the paucity of timely and high-quality immunization data. Indeed, in many settings, existing data systems can rarely guarantee the generation of timely and good-quality data at all health system levels - a weakness that can have dire consequences on various aspects of immunization [5, 6]. For instance, inadequate immunization data quality can lead to missed opportunities to identify under-immunized or zero-dose children, poor defaulter tracking, and inaccurate vaccine coverage estimation [6]. This, in turn, could undermine national and international investments and increase the risk of vaccine-preventable diseases (VPD) by failing to identify populations with low vaccination coverage [7].

Gavi, The Vaccine Alliance made data quality a strategic focus area in 2015, highlighting quality immunization data as a ‘critical enabler’ to achieving immunization targets [8, 9]. Yet, poor immunization data quality continues to be a pervasive problem across many Gavi-eligible countries. For example, a study assessing the validity of reported vaccination coverage in 45 Gavi-eligible countries found that 75.5% of officially reported coverage figures from administrative sources were at least 10% higher than national survey estimates [10]. Similarly, in a study evaluating the quality of immunization monitoring systems in 27 Gavi-supported countries (including Cameroon), Ronveaux *et al* in 2005 reported that only 60% of health units had data recording forms (immunization registers and tally sheets) available for a whole year, and that only 59% of health workers completed a vaccination card properly [11]. Similarly, a study carried out in North-West Ethiopia noted that nearly half of all surveyed health facilities did not have standardized immunization registers and tally sheets available [12]. Lack of critical immunization data collections tools as well as inadequate data recording practices invariably affects a country’s immunization coverage estimates.

In Cameroon, remarkable progress has been made to improve national immunization coverage, since the inception of Cameroon’s Expanded program on immunization [13]. However, despite making noteworthy progress, coverage rates for many antigens still fall below set targets [14, 15]. A few studies have attempted to examine the performance of different components of Cameroon’s immunization system [16–21], exploring possible barriers and facilitators to optimal immunization coverage. However, to the best of our knowledge, there is no published study evaluating the data management practices of frontline immunization staff in Cameroon. This study was, therefore, designed to assess the data management practices of immunization staff in healthcare facilities, and it unveiled several barriers to high-quality and timely immunization data in Cameroon.

## Materials and methods

### Study design

This was a descriptive cross-sectional facility-based study carried out as part of a national baseline assessment to identify and describe factors contributing to declining immunization coverage in Cameroon.

### Study setting

The study was conducted in Cameroon, a country located in Central Africa, at the Gulf of Guinea, with a population of approximately 28 million inhabitants, and covering a total surface area of 475,440 km^2^ [22]. The country is divided into 10 administrative regions, namely Adamawa (AD), Centre (CE), East (ES), Far-North (EN), Littoral (LT), North (NO), North-West (NW), West (OU), South (SU) and South-West (SW) regions [23].

Cameroon’s health sector is organized into three main levels (central, intermediate, and peripheral), with each level having specific competencies, administrative, health and dialogue structures [24]. The central level is headed by the ministry of public health and focuses mainly on the development of policies, strategies, and coordination. The intermediate level is headed by the 10 regional delegations and this level provide technical support to the country’s 189 health districts. The health districts, in turn, are mainly responsible for delivering primary health care services to the Cameroonian population. Preventive services such as immunization services are incorporated into all levels of the health system [25].

Immunization performance monitoring is achieved through a hierarchical administrative data monitoring system. Focal EPI staff at health facilities aggregate data on monthly tally sheets into paper-based monthly activity report, which are then transmitted to the district data management team for entry into the District Health Information System (DHIS2). Immunization data in the DHIS2 are then aggregated at a regional level before transmission to national level for national estimates of coverages for various indicators [26].

### Sampling

A minimum sample size of 145 health facilities was estimated using the following formula and parameters.

$$N = {\rm{ }}\left[ {DEFF{\rm{ }}x{\rm{ }}Z2{\rm{ }}x{\rm{ }}p\left( {1 - p} \right)} \right]{\rm{ }}/{\rm{ }}E2$$

Design effect (DEFF) of 1.5, Critical value (Z) of 1.96 for a confidence level of 95%, estimated proportion of outcome (p) = 50%, and margin of error (E) of 10%.

Multistage sampling was used after allocating the number of districts and health facilities to be selected. The number of districts was allocated in proportion to the total number of districts per region, in the ten regions. Then, the number of urban and rural districts were assigned within each region based on the region-specific breakdown and health facilities allocated across regions in proportion to the national distribution.

The districts were then randomly selected within specified region urban or rural strata in the first stage and health facilities were randomly selected within the identified rural/urban districts in the second stage. This was done while ensuring that the same number of facilities was selected within each district in a region.

### Study procedures

#### Trainings

Trainings were carried out in all regions by members of the baseline assessment management team to provide regional supervisors and assessors with the necessary knowledge and skills to undertake the baseline assessment. Trainings lasted a minimum of two days and consisted of theoretical presentations and practical sessions on data collection, entry, and transmission processes.

#### Data collection

Data collection plans for targeted districts and health facilities were developed by the baseline assessment management team and the regional EPI teams prior to data collection. To prevent unproductive visits during data collection, assessors contacted the health facilities via phone to remind them of planned visits. Upon arrival in facilities, the purpose of the assessment was explained to the facility head, or their representative and the health facility focal EPI staff interviewed. Additionally, an ongoing fixed-post vaccination session was observed, and vaccination data collection tools were physically inspected.

### Study tools

The tools used for this study included health facility questionnaire, (which was designed to assess immunization data collected and availability of data collection tools) and an immunization service observation guide and checklist, (which comprised of several prompts to assess data management practices of immunization staff during vaccination sessions, and the data recorded in core data collection tools). These study tools were developed in English and French, and pre-tested in four facilities in the Centre region before being used to collect data for the study.

### Data management and analysis

A comprehensive database was built, pre-tested and validated by an expert data manager prior to data entry. Each assessor entered data from the filled questionnaires and observation forms into the database and transmitted the files to a secure server within three days of data collection. Data were exported and cleaned in Microsoft Excel 2016 and analyzed with Stata 13 software (StataCorp. 2013. Stata Statistical Software: Release 13. College Station, TX: StataCorp LP.). Frequencies and proportions were used to summarize variables, with health facility used as the unit of analysis. Since districts and facilities were sampled proportional to national distributions, no post-stratification weights were applied.

### Operational definitions

- **Data management practices**: This included data collected, availability and utilization of data collection tools, data consistency across collection tools and data recording practices during vaccination sessions.
- **Vaccination staff** (Used interchangeably with immunization staff, vaccine-providers, vaccinators): This included all health staff working in the immunization unit.
- **Completely filled data collection tool**: All required columns of the immunization tool were filled, for one month until the time of assessmentTo examine this data on completeness for five routine immunization tools was assessed: Immunization register, tally sheets, monthly reporting form, temperature chart, stock ledger.

### Administrative approval

Administrative approval was obtained from the ministry of public health and all ten regional delegations of public health prior to commencement of the baseline assessment.

### Ethical considerations

This study was conducted in compliance with the Helsinki Declaration and all applicable national laws and institutional rules ethical approval was granted by the Cameroon National Ethics Committee for Human Health Research prior to study commencement.

## Results

Table 1 presents the general characteristics of surveyed facilities. Overall, 265 health facilities in 68 health districts were surveyed, with slightly over half (53%) of these facilities located in rural areas. At least two staff worked in the immunization service in most (74%) of these facilities. Over two-thirds (67%) had staff who have never received training on immunization (Table 1). We also noted great regional variations in trainings, with 93% and 100% surveyed in the South region and East regions lacking a single staff who has received basic training on immunization, respectively.

**Table 1:** General characteristics of surveyed health facilities

Regions	AD	CE	ES	EN	LT	NO	NW	SU	SW	OU	NAT
**Distribution of health facilities**											
Rural (N)	8	22	14	10	12	8	26	19	8	13	**140**
Urban (N)	3	34	7	6	22	8	9	13	6	17	**125**
Total (N)	11	56	21	16	34	16	35	32	14	30	**265**
**% of HCW working in immunization per health facility**											
Less than 2	11	9	23	4	16	0	24	31	33	7	**16**
2 to 5	67	86	65	91	77	83	61	54	55	90	**74**
More than 5	22	5	12	5	7	17	15	15	12	3	**10**
**% of trained HCW per health facility**											
None	78	66	100	68	59	84	49	93	76	61	**67**
1 to 2	22	30	0	23	26	17	32	8	12	23	**22**
3 to 5	0	2	0	9	13	0	15	0	6	13	**7**
More than 5	0	2	0	0	13	0	5	0	6	3	**4**

NAT National, AD Adamawa, CE Center, ES East, EN Extreme North, LT Littoral, NO North, NW North West, SU South, SW South West, OU West, HCW Health care worker, HF Health Facility

### Data collected by health facilities

Table 2 summarizes the practices of healthcare workers with regards to documenting data on vaccine administration. As illustrated 92% of facilities systematically collect vaccine administration data (filling of tally sheets, immunization registers and vaccination cards) at national level. At regional level, marked variations were observed. As shown in Table 2, about a quarter of (24%) of surveyed facilities in the East and South-West Regions did not document vaccine administration data. Only 65% and 61% of health facilities collected stock levels and vaccine wastage respectively. As regards temperature monitoring, most (79%) facilities recorded vaccine storage temperatures at the time of assessment.

**Table 2:** Availability and utilization of data collection tools (%)

Regions	AD	CE	ES	EN	LT	NO	NW	SU	SW	OU	NAT
**Data collected by health facilities**											
Vaccinations	89	100	76	100	100	92	85	92	76	100	**92**
Stock levels	89	52	35	86	74	83	76	23	70	61	**65**
Vaccine storage temperatures	89	75	65	91	87	75	83	46	73	94	**79**
Vaccine wastage	56	50	41	77	84	50	71	38	64	55	**61**
**Availability of data collection tools**											
Tally sheets	89	91	65	100	100	92	85	69	45	94	**84**
Immunization register	78	96	76	100	97	92	85	100	48	94	**87**
Monthly reporting forms	89	93	65	100	94	92	85	85	48	94	**85**
Vaccination cards	11	32	53	86	58	17	56	23	42	19	**43**
Temperature monitoring sheet	89	63	71	91	87	83	83	54	45	90	**74**
Repair and maintenance forms	11	5	12	9	16	0	15	0	3	6	**8**
Stock cards/register	89	45	29	86	71	58	73	31	45	55	**57**
AEFI forms	33	34	35	45	19	0	51	23	30	23	**32**
Lost to follow-up registers	33	11	6	23	29	17	41	15	21	32	**23**
**Effective use of tools (%of health facilities with completely filled tool)**											
Immunization register	78	66	59	73	77	67	56	62	48	55	**63**
Tally sheets	0	5	0	0	0	0	78	0	45	0	**19**
Monthly reporting form	0	4	0	0	0	0	51	0	36	0	**13**
Temperature chart	44	39	12	64	58	33	68	23	42	19	**43**
Stock ledger	44	29	6	36	29	58	44	8	39	19	**31**
**Data consistency across data recording tools**											
Health facilities with discrepancies for Penta3 across different tools	78	57	41	41	81	58	39	85	24	74	**53**

*NAT: National, AD Adamawa, CE Center, ES East, EN Extreme North, LT Littoral, NO North, NW Northwest, SU South, SW South West, OU West, AEFI Adverse event following immunization

### Availability and utilization of data collection tools

At national level, majority of health facilities had core vaccination data collection tools, including tally sheets (84%), registers (87%), reporting forms (85%). Conversely, only 43% of facilities had vaccination cards, 8% had maintenance registers and 32%  had stock cards. Regarding the use of these tools, immunization registers were filled in only 63% of health facilities and tally sheets just in 19% of facilities. Similarly, only 13% of facilities had updated and fully filled monthly report forms. These findings are shown in Table 2.

### Data consistency across recording tools

Discrepancies for the same data was noted across three core data collection tools assessed (tally sheets, immunization registers and monthly reporting forms). The Pentavalent 3 value from the tally sheets and monthly reporting forms was different in over half (53%) of the health facilities. This was observed across all regions and the highest percentage of health facilities with inconsistent data across sources was noted in the South (85%) and Littoral (81%) regions, as shown in Table 2.

### Data recording practices during immunization sessions

Immunization registers and tally sheets were present at the vaccination site in up to 90% of facilities. Similarly, in most (85%) health facilities, immunization acts were documented in registers, tally sheets and vaccination cards as recommended, with regional variations (ranging from 73% in the East region to 100% in the North-West and South-West regions). Conversely, only 11% of health facilities adhered to the recommendation of filling the tally sheet as children were vaccinated. Up to 51% of these facilities rather filled the sheets before vaccination, and 25% did so after vaccination as illustrated in Table 3.

**Table 3:** Data recording practices during vaccination sessions (%)

Regions	AD	CE	ES	EN	LT	NO	NW	SO	SW	OU	NAT
**Immunization registers and tally sheets were present at the vaccination site**											
Yes	89	88	93	79	94	88	94	85	95	91	**90**
No	11	12	7	21	6	12	6	16	5	9	**10**
**Vaccinations recorded in vaccination registers, tally sheets and vaccination cards**											
Yes	89	80	73	75	94	82	100	77	100	84	**85**
No	11	20	27	25	6	18	0	23	0	16	**15**
**Time points when the tally sheet was filled out**											
Before vaccination	78	46	40	29	71	29	59	38	64	48	**51**
During vaccination	0	9	0	29	12	18	21	0	5	6	**11**
After vaccination	0	25	33	25	15	35	21	38	27	29	**25**
Never	22	19	27	17	3	18	0	23	5	16	**13**

*NAT: National, AD Adamawa, CE Center, ES East, EN Extreme North, LT Littoral, NO North, NW Northwest, SU South, SW Southwest, OU West

## Discussion

The main objective of this study was to assess the data management practices of immunization staff at health facility level, in a bid to unveil potential barriers to vaccine data quality in Cameroon. To the best of our knowledge, this is the first published study examining the data management practices of vaccination staff on a national scale in Cameroon. We noted several unsatisfactory data collection and management practices in a good proportion of facilities including ineffective use of data collection tools, filling tally sheets before or after vaccination, sub-optimal collection of data on some key immunization indicators. This study also found that the unavailability of core data recording tools was not uncommon and could contribute to substandard data collection and eventually immunization data quality.

We noted various gaps when it comes to recording immunization data at facility level. In our study we found that about one in every ten (8%) of facilities did not collect data on vaccine administration; about one in three (35%) did not collect data on stock levels and about two in five (21%) did not record vaccine storage temperatures and vaccine wastage. Our findings lend support to those documented in multiple settings [7, 9, 10, 27]. As documented elsewhere, several technical, organizational and behavioral factors may explain the root causes of the observed deficits in this study, including gaps in health provider capability and motivation, poor supportive supervision, unavailability or complexity of data recording tools, lack of performance-based targets, and interrupted regular feedback [12, 28]. Deficits in capacity and capability could explain the gaps in indicators like vaccine wastage, which may be relatively more complex to compute. The observed data collection gaps could also be linked to the limited availability of some data recording tools like vaccination cards, maintenance registers and stock cards, which were lacking in 57%, 92% and 43% of surveyed facilities, respectively (Table 2).

The availability of immunization registers (87%) and tally sheets (83%) in our study is higher than what was reported by Kefiyalew et al. (available in 43–50% of facilities) in Ethiopia [12] and Ronveaux et al. [11] in 27 GAVI-supported countries, including Cameroon (available in 60% of facilities). These differences could be because Kefiyalew’s study was carried out only in one health district while Ronveaux’s study assessed availability of tools over a one-year window, unlike ours which assessed only during the study period. Difference could also reflect improvements by Cameroon’s EPI, to ensure that these critical data collections are available at facility level. Such improvements were, to a large extent, driven by governments efforts to introduce new vaccines such as IPV, combined measles vaccines, HPV etc., which are always accompanied by updating and distribution of various data collections tools. Other organizations, such as the Clinton Health Access initiative and Unicef have also supported the government to provide facilities with registers.

We also found that even when data recording tools were available and used, they were ineffectively used and incompletely filled in a good proportion of facilities, further aggravating data quality problems at facility level. This finding is comparable to that of Wallace *et al.* (2017) in Nigeria, who reported that only 51–55% assessed facilities stock records with sufficient data to calculate vaccine wastage rates [29]. Additionally, our study noted inconsistencies for the same data recorded across different core data recording tools. For instance, the Pentavalent 3 value from the tally sheets and monthly reporting forms was discrepant in over half (53%) of the health facilities. This finding is slightly lower compared that reported by Akerele et al. (2020) in neighboring Nigeria, who showed that Pentavalent 3 values between the health facility monthly summary form and the national health management information systems monthly summary form were discrepant in 67% assessed facilities in Kano State [27]. These gaps can be corrected by health worker training on immunization data tools and regular supportive supervisory visits, interventions which have been shown by Akerele et al. (year) as important determinants of good data consistency.

In our study we also identified some data management practices during vaccination sessions which could contribute to incomplete, or inaccurate or inconsistent data. Just over a tenth (11%) of health facilities adhered to the recommendation of filling the tally sheet as children are being vaccinated, instead of filling them before or after vaccination. This could lead to either under-reporting or over-reporting of vaccinated children, resulting into poor data quality which could impact in-country and external investments, and frustrate efforts in reaching zero-dose children and under-immunized children. This further reemphasizes the need for staff capacity building, supportive supervision, and standardization of data recording processes.

While interpreting the findings of our study, some limitations have to be considered. Our study may be biased by the Hawthorne effect because EPI personnel were aware of being observed and may have modified their behaviors during the study period. Secondly, data management practices were assessed at health facility level, thus individual level variations at health facility level could be missed.

## Conclusion

This study brings to light several unsatisfactory data management practices of vaccination staff in Cameroon, including ineffective use of data collection tools, filling tally sheets before or after vaccination, sub-optimal collection of data on some key immunization indicators. These, coupled with unavailability of key data recording tools, could hinder the generation of timely, high-quality data, and ultimately affect decision making and delivery of immunization services. There is thus a crucial need to standardize data recording processes, systematically build data management competencies of health facility EPI staff in Cameroon, while building policies and systems that ensure constant availability of data recording tools.

## Data Availability

Data used for this research are available from the corresponding author upon reasonable request.

## Declarations

### Administrative Approval

Administrative approval was obtained from the ministry of public health before data collection. Additionally, written approvals were also obtained from all regional delegations of public health, who in turn issued administrative letters to district heads requesting full support for the data collection process.

## List of abbreviations

CHAI: Clinton Health Access Initiative
EPI: Expanded Program on Immunization
HCW: Healthcare workers
HF: Health facility
MCV: Measles containing vaccine
MOV: Missed Opportunities for Vaccination
VPD: Vaccine-Preventable Diseases
WHO-MDVP: WHO Multi-dose Vaccine Policy
